# Supplementary material for: Silver Nanoparticles Densely Grafted with Nitroxides as a Recyclable Green Catalyst in the Selective Oxidation of Alcohols
Source: Nanomaterials (Basel). 2022 Jul 24;12(15):2542. doi: 10.3390/nano12152542 (PMC9330881; doi:10.3390/nano12152542)
Supplement: Supplementary file 1 [file nanomaterials-12-02542-s001.zip › nanomaterials-1804808-supplementary.pdf]

Supporting Information

# Silver nanoparticles densely grafted with nitroxides as a recyclable green catalyst in the selective oxidation of alcohols

Agnieszka Krogul-Sobczak[a], Natalia Pisarek[a], Piotr Cieciórski[a], Elżbieta Megiel*[a]

[a] Faculty of Chemistry, University of Warsaw, Ludwika Pasteura 1, 02-093 Warsaw, Poland.

E-mail: e.megiel2@uw.edu.pl

**Figure S1** ESR spectrum of obtained N-AgNPs and ligand (DiSS). All samples were prepared in acetone as a solvent at 293K.

**Figure S2** XPS survey spectrum of synthesized N-AgNPs.

**Figure S3** XPS spectra: C 1s a) and O 1s b) of N-AgNPs. The values of BDE are presented in brackets.

**Figure S4** TGA curves of obtained N-AgNPs and ligand (DiSS) used for their preparation.

**Table S1.** Oven temperature values in GC analyses.

**Table S2.** The yields of oxidation of benzyl alcohol to benzaldehyde in the presence of TEMPO determined for six repeated experiments under conditions applied in all presented in this paper reactions, and the standard deviation calculated from these results.[a]

**Table S3.** Results of catalytic oxidation of selected alcohols using TEMPO as an organocatalyst with various molar ratios and reaction times.[a]

**Table S4.** Oxidation of benzyl alcohol in the presence of N-AgNPs depending on time.[a,b]

**Table S5.** The reusability of N-AgNPs in catalytic oxidation of 4-pyridinemethanol.[a]

**Table S6.** The comparison of selected Cu/TEMPO Systems in the oxidation of benzyl alcohol with results obtained in this work.

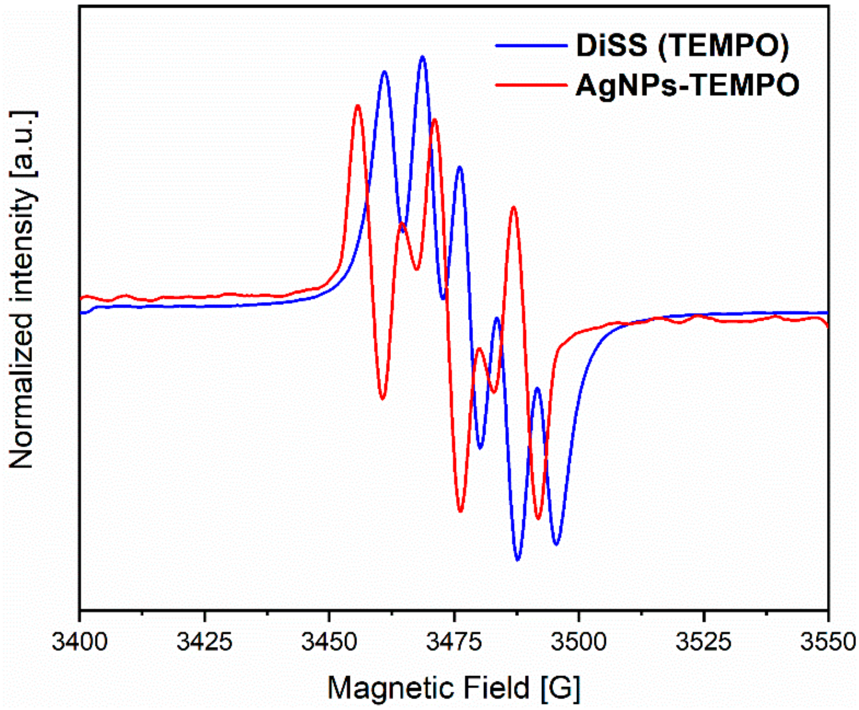


**Figure S1** ESR spectrum of obtained N-AgNPs and ligand (DiSS). All samples were prepared in acetone as a solvent at 293K.

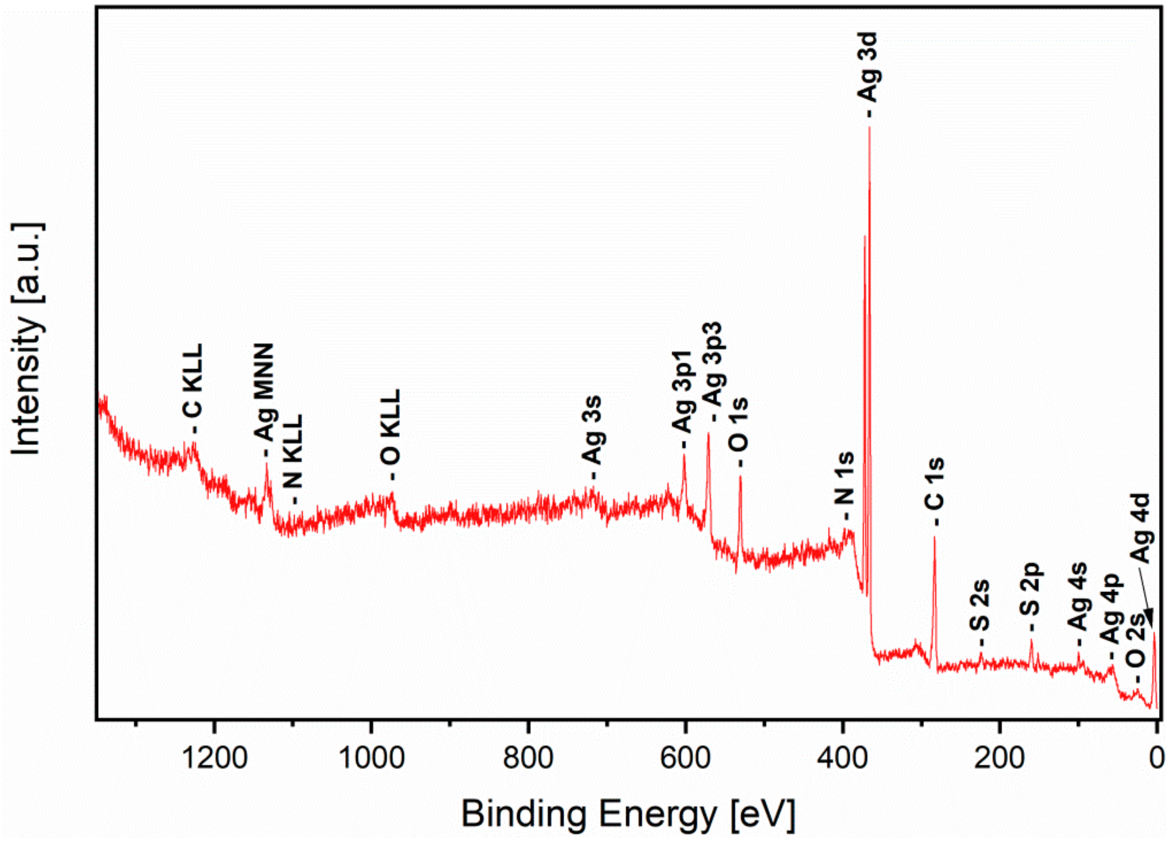


**Figure S2** XPS survey spectrum of synthesized N-AgNPs.

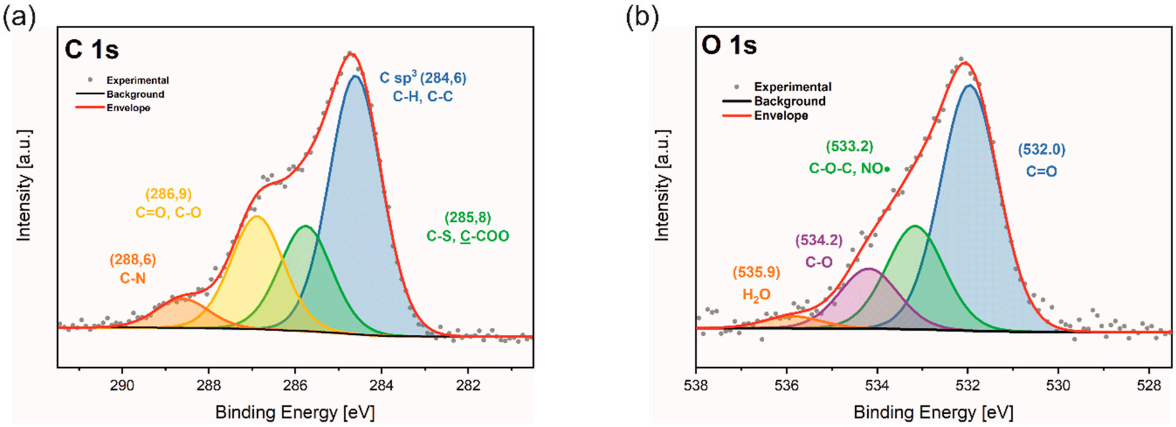


**Figure S3** XPS spectra: C 1s (a) and O 1s (b) of N-AgNPs. The values of BDE are presented in brackets.

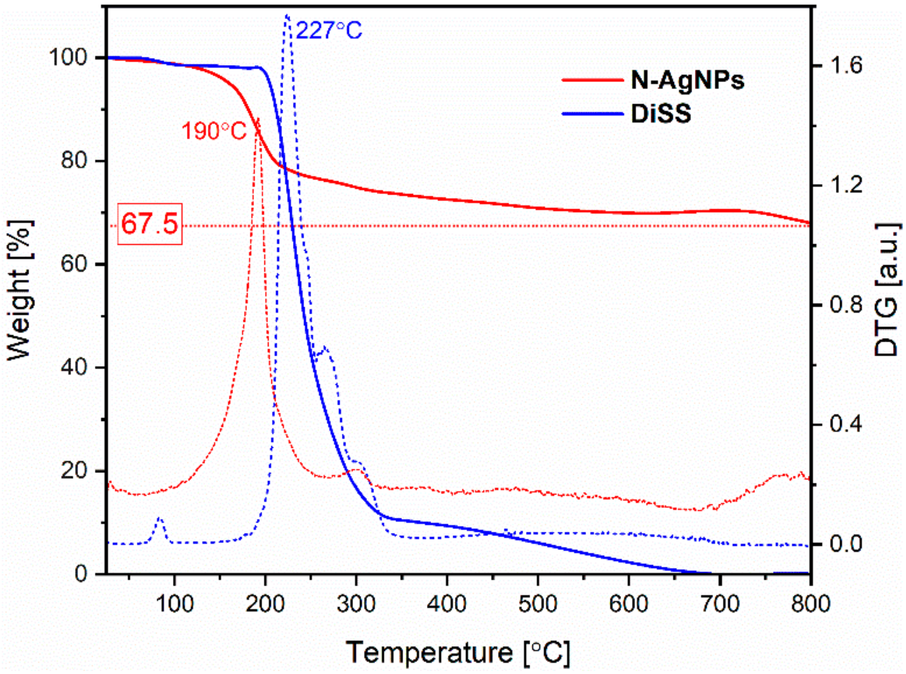


**Figure S4** TGA curves of obtained N-AgNPs and ligand (DiSS) used for their preparation.

**Table S1** Oven temperature values in GC analyses.

| **No** | **substrate / product** | $T_{oven}$ |
|---|---|---|
| 1 | benzyl alcohol / benzyl aldehyde | 100 |
| 3 | 4-pyridinemethanol / 4-pyridinaldehyde | 180 |
| 5 | furfuryl alcohol / furfuryl aldehyde | 80 |
| 7 | heptanol / heptanal | 100 |
| 9 | 1-phenylethanol / acetophenone | 100 |

**Table S2** The yields of oxidation of benzyl alcohol to benzaldehyde in the presence of TEMPO determined for six repeated experiments under conditions applied in all presented in this paper reactions, and the standard deviation calculated from these results.[a]

| Reaction No | Yield [%] |
|---|---|
| 1 | 92.5 |
| 2 | 100 |
| 3 | 100 |
| 4 | 99.1 |
| 5 | 100 |
| 6 | 92,0 |
| mean | 97.3 |
| error | +/– 3.9 |

[a] Reaction conditions: alcohol (1 mmol, 2M in MeCN), Cu(OTf) (3 mol %), bipy (3 mol %), TEMPO (1,12 mol %), NMI (6 mol %), atm. air, rt, time 2,5 h. Yields were determined by GC analysis, based on the ratio of product/(product + substrate).

**Table S3.** Results of catalytic oxidation of selected alcohols using TEMPO as an organocatalyst with various molar ratios and reaction times.[a]

| Entry | Substrate / Formula | Product / Formula | TEMPO [mol%] | Time [h] | Yield [%] | TON[b] |
|---|---|---|---|---|---|---|
| 1 | | | 3 | 1 | 78 | 26 |
| 2 | benzyl alcohol | benzaldehyde | | 2.5 | 100 | 33 |
| 3 | 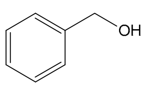 | 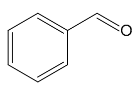 | 1.12 | 1 | 66 | 59 |
| 4 | | | | 2.5 | 100 | 89 |
| 5 | | | 0.56 | 2.5 | 70 | 125 |
| 6 | | | 3 | 2 | 73 | 24 |
| 7 | 4-pyridinemethanol | 4-pyridinecarboxaldehyde | | 2.5 | 80 | 27 |
| 8 | 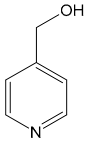 | 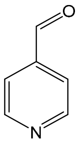 | | 3 | 86 | 29 |
| 9 | | | 1.12 | 2 | 70 | 63 |
| 10 | | | | 2.5 | 79 | 71 |
| 11 | | | | 3 | 83 | 74 |
| 12 | | | 3 | 1 | 61 | 20 |
| 13 | | | | 1.5 | 90 | 30 |
| 14 | furfuryl alcohol | furfural | | 2 | 100 | 33 |
| 15 | | | | 2.5 | 100 | 33 |
| 16 | 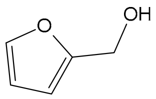 | 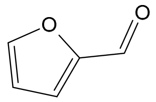 | 1.12 | 1 | 40 | 36 |
| 17 | | | | 1.5 | 65 | 58 |
| 18 | | | | 2 | 95 | 85 |
| 19 | | | | 2.5 | 100 | 89 |
| 20 | | | 3 | 1 | 47 | 16 |
| 21 | | | | 2 | 82 | 27 |
| 22 | 1-phenylethanol | acetophenone | | 2.5 | 86 | 29 |
| 23 | | | | 3 | 90 | 30 |
| 24 | 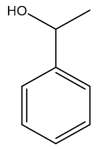 | 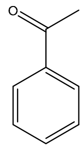 | | 4 | 95 | 32 |
| 25 | | | | 5 | 97 | 32 |
| 26 | | | 1.12 | 2 | 53 | 47 |
| 27 | | | | 2.5 | 57 | 51 |
| 28 | | | | 3 | 61 | 54 |
| 29 | | | 3 | 2 | 75 | 25 |
| 30 | | heptanal | | 2.5 | 91 | 30 |
| 31 | 1-heptanol | | | 3 | 97 | 32 |
| 32 | 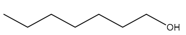 | 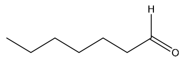 | 1.12 | 2 | 76 | 68 |
| 33 | | | | 2.5 | 82 | 73 |
| 34 | | | | 3 | 85 | 76 |

[a] Reaction conditions: alcohol (1 mmol, 2M in MeCN), Cu(OTf) (3 mol%), bipy (3 mol%), NMI (6 mol%). Yields were determined by GC analysis (products were not isolated) based on the product/(product + substrate). [b] Turnover number expressed in a mmol aldehyde or ketone/mmol of TEMPO

**Table S4** Oxidation of beznyl alcohol in the presence of N-AgNPs depending on time.[a,b]

| Entry | No cycle | Time [h] | Yield[b] [%] | S[c] [%] | TON[d] |
|---|---|---|---|---|---|
| 1 | I | 2 | 91 | 97 | 108 |
| 2 | I | 2.5 | 96 | 96 | 114 |
| 3 | II | 2.5 | 85 | 96 | 101 |
| 4 | II | 3 | 92 | 96 | 110 |
| 5 | II | 4 | 96 | 96 | 114 |
| 6 | III | 4 | 90 | 96 | 107 |
| 7 | IV | 4 | 81 | 96 | 97 |
| 8 | V | 4 | 82 | 96 | 98 |
| 9 | VI | 4 | 78 | 97 | 93 |
| 10 | VII | 4 | 67 | 95 | 80 |

[a] Reaction conditions: benzyl alcohol (1 mmol, 2M in MeCN), Cu(OTf) (3 mol%), bipy (3 mol%), N-AgNPs (9 mg = the amount containing 0.0084 mmol of organic ligand in N-AgNPs), NMI (6 mol%), air atm., rt, time 2-4 h. After each cycle contents of the tube were centrifuged (6000 rpm, 6 min) and left in MeCN for one night. [b] Yield of main product was determined by GC analysis, based on ratio of (main product)/(all products + starting material). [c] Selectivity towards main product was determined by GC analysis, based on ratio of (main product)/(all products). [d] Turnover number expressed in [(mmol of main product) × (mmol of catalytically active form of organic ligand in N-AgNPs)$^{-1}$].

**Table S5.** The reusability of N-AgNPs in catalytic oxidation of 4-pyridinemethanol.[a]

| **No cycle** | I | II | III | IV | V | VI | VII | VIII | IX | X | XI | XII | XIII | XIV |
|---|---|---|---|---|---|---|---|---|---|---|---|---|---|---|
| **Yield [%]** | 79 | 73 | 65 | 58 | 56 | 62[b] | 60 | 64[b] | 59 | 54 | 55 | 40 | 48 | 46 |
| **No cycle** | XV | XVI | XVII | XVIII | XIX | XX | XXI | XXII | XXIII | XXIV | XXV | XXVI | XXVII | |
| **Yield [%]** | 48[b] | 44 | 28 | 21 | 27 | 33[b] | 27 | 25 | 25 | 24 | 24 | 21 | 20 | |

[a] Reaction conditions: 4-pyridinemethanol (1 mmol, 2M in MeCN), Cu(OTf) (3 mol%), bipy (3 mol%), N-AgNPs (the amount corresponding to 0.0084 mmol of organic ligand), NMI (6 mol%), air atm., rt, time 2.5 h. Yields of aldehydes were determined by GC analyses based on the product/(product + substrate) ratio. After each cycle, the tubes' content was centrifuged (6000 rpm, 6 min) and left in MeCN for one night. [b]After the previous cycle, centrifuged nanoparticles were left in MeCN for two nights.

**Table S6.** The comparison of selected Cu/TEMPO Systems in the oxidation of benzyl alcohol with results obtained in this work (references are placed in the main text).

| | **This work** | **Ref. 15** | **Ref. 16** | **Ref. 16** | **Ref. 17** | **Ref 49** | **Ref 20** | **Ref 20** |
|---|---|---|---|---|---|---|---|---|
| **Amount of TEMPO** | N-AgNPs 0.84 mol% (9 mg containing 0.0084 mmol of TEMPO) | 7.5 mol% TEMPO | 5 mol% TEMPO | 5 mol% TEMPO | 5 mol% TEMPO | 3 %mol TEMPO | Silica-TEMPO (100 mg containing 0.02 mmol of nitroxide radicals) | Ferrite-TEMPO (200 mg containing 0.018 mmol of nitroxide radicals) |
| **Source of Cu** | $(bpy)Cu^{I}$ | $CuBr_2$ | $CuBr_2$ | $Cu(ClO_4)_2$ | $(bpy)Cu^{I}$ | $CuBr_2$ | $Cu(NO_3)_2 \cdot 3H_2O$ | $Cu(NO_3)_2 \cdot 3H_2O$ |
| **Yield** | 96% | 100% | 83% | 90% | 95% | 78% | 90% | 44% |
| **TON** | 114 | 13 | 17 | 18 | 19 | 26 | 45 | 24 |
